# MiniQEEG and Neurofeedback in Diagnosis and Treatment of COVID-19-Related Panic Attacks: A Case Report

**DOI:** 10.3390/brainsci12111541

**Published:** 2022-11-14

**Authors:** Marta Kopańska, Agnieszka Dejnowicz-Velitchkov, Paulina Bartman, Jacek Szczygielski

**Affiliations:** 1 Department of Pathophysiology, Institute of Medical Sciences, University of Rzeszow, 35-959 Rzeszow, Poland; 2 ADEA, Ltd., Biofeedback Center, 1000 Sofia, Bulgaria; 3 Students Science Club “Reh-Tech”, Institute of Medical Sciences, University of Rzeszow, 35-959 Rzeszow, Poland; 4 Department of Neurosurgery, Institute of Medical Sciences, University of Rzeszow, 35-959 Rzeszow, Poland; 5 Department of Neurosurgery, Faculty of Medicine, Saarland University, 66123 Saarbrücken, Germany

**Keywords:** COVID-19, quantitative electroencephalography, neurofeedback, panic attacks

## Abstract

Background: Both the global COVID-19 pandemic situation, as well as the current political situation in Eastern Europe may exacerbate anxiety and contribute to stress-related disorders such as panic disorder. Electroencephalography (EEG)-based neurofeedback provides both assessment of the subject’s brainwave activity as well as the possibility of its therapeutic correction. It is possible that it can be implemented as an auxiliary treatment in panic disorders of different origin. The aim of this feasibility study was to demonstrate (both short- and long-term) effectiveness of neurofeedback therapy in a patient with previously diagnosed panic attacks, related to fear of COVID-19 infection. Methods: We report the case study of a 47-year-old man affected by panic attacks, related to his profound, constant fear of COVID-19 infection and its sequelae. For the initial diagnostic workup, several clinical and research tools were used: 1. Baseline psychological exam; 2. Anxiety—targeted interview performed by miniQEEG therapist; 3. Analysis of previous clinical test results (EEG record/lab blood test); and 4. The miniQEEG exam (central strip recording Cz-C3-C4), The patient was subjected to regular EEG Neurofeedback sessions for two consecutive months. After completing the treatment, follow-up tests, as listed above were repeated immediately after completing the whole treatment program, as well as 1 and 2 years later. MiniQEEG results were compared with healthy control (age-matched male subject not affected with panic attacks) and evaluated over the time that the subject was involved in the study. Results: Initially, the patient was suffering from severe panic attacks accompanied by vegetative symptoms and from destructive and negative thoughts. After 8 consecutive weeks of treatment encompassing sixteen QEEG neurofeedback training sessions (each lasting 30 min), a subjective improvement of his complaints was reported. More importantly, QEEG records of the patient also improved, approximating the pattern of QEEG recorded in the healthy control. Conclusion: In this single case-based feasibility analysis, we demonstrate that systematic application of QEEG-Neurofeedback may result in manifest and durable therapeutic effect. Of note, use of this treatment may be a valuable option for patients with panic attack/panic disorder, especially if related to the psychological burden of the COVID-19/war era. Future studies on a larger patient population, especially with a longitudinal/prospective design, are warranted.

## 1. Introduction

Panic disorder (PD) is one of the most common disturbances of mental health encountered in clinical practice. This condition is characterized by excessive alertness and activity leading to significant behavioral, cognitive and emotional symptoms potentially affecting everyday functioning [1]. Based on the definition in the DSM-5, panic disorder (PD) is an anxiety disorder characterized by signs arising from the circulatory, respiratory, digestive, and autonomic nervous systems [2]. Further, panic attacks (PAs) are a key feature in primary PD diagnostic workup and they pose a clinically significant indicator both for comorbidity and for the risk of developing the full-blown disorder [3]. They can be identified by repetitive and unexpected spells of fear or anxiety that have a rapid onset and usually reach their maximum intensity within 10 min involving at least four or more of typical components. These symptoms may include palpitations and accelerated heart rate, chest pain, sweating, trembling or shaking, feeling of choking, dizziness, unsteadiness, being lightheaded or fainting, heat sensations, nausea or abdominal distress and sensations of shortness of breath [4,5]. Further on, panic attack symptoms may include fearful cognitions, such as the fear of collapse, going mad or dying, and feeling of unreality (derealization) or being detached from oneself (depersonalization). Panic attacks are quite common in the general population [6]. However, in PD patients, their persistence, the high lifetime number of attacks, and the cumulative number of years with attacks may define their debilitating character [7,8], so that patients with anxiety disorders are burdened with a higher rate of unemployment and may require hospitalization more often than the general population [9,10].

Among many strategies for PD management [11], quantitative electroencephalogram (QEEG) has been recently recognized as a useful tool in planning and monitoring the therapy for anxiety and related disturbances. Compared to other electrophysiological and functional brain imaging techniques, QEEG seems to be highly reliable, repeatable and observer-independent, enabling electrophysiological screening at a low cost [12,13,14]. Compared with plain EEG, QEEG offers a better possibility of visualizing the results and of analyzing them [15]. The obvious advantage is also the non-invasiveness and compact design of the QEEG appliance, enabling the survey of the electrical brain activity both at rest and during various activities [16,17]. QEEG may be considered as a useful add-on in completing the diagnosis of PD or even in predicting the risk of disease exacerbation or serious consequences of a chronic anxiety state for physical health (including heart attack or stroke) [18,19,20,21].

Notably, the advantages of QEEG are not limited to its diagnostic use. Coupling EEG or QEEG records with electronic devices and visualization tools provides the individual with an insight into fluctuations/changes in their own electrical activity in their brain (feedback given in a visual or acoustic form). More so, the given person may be trained to (at least partially) control their own EEG rhythm (EEG-neurofeedback) [22]. Through EEG-neurofeedback, the patient learns to consciously modify the physiological function that normally is out of voluntary control e.g., brain waves.. The essence of QEEG-neurofeedback is to model electric activity in the brain, based on the ability of neurons to carry out and to maintain the modification of the pattern of their electrical activity if repeatedly conditioned by attempts at intrinsic regulation [23]. The previous evidence on EEG-neurofeedback use in different pathopsychological conditions (mostly behavioral disturbances) is vast [21,22]. Nevertheless, available data on its application in PD/anxiety condition (particularly those related to recent angst-inducing global events, including the COVID-19 pandemic), are somewhat limited. For this reason, we were interested to see if biofeedback therapy based on a QEEG analysis may be performed in a patient with panic attacks, bringing any benefit (preferably of enduring character).

The aim of this single case analysis is to determine if the QEEG-based neurofeedback therapy is suitable to treat the condition, with main complaints and clinical demonstration resulting from previously diagnosed PD. As the contemporary issue, the current COVID-19 pandemic as the trigger for the emergence of PD, was considered.

## 2. Materials and Methods

This feasibility single-case study was conducted with a 47 years old male, with an academic degree, working in a management position. He was married for 15 years, raising two children of early school age. There was no history of mental or neurological diseases in the family. Before the study-related examination he was not treated before by psychologist or psychiatrist. The panic attacks appeared first in February 2020. They were related to the COVID-19 positive test, with subsequent aggravating concerns about the subject’s health condition and employment. In the beginning he did not associate his symptoms (a racing heartbeat, sweating, chest pain, shortness of breath and general anxiety) with psychological problems; for this reason, he sought the advice of the general practitioner. After a thorough clinical workup, physical reasons for the complaints could be ruled out and the patient was referred to a psychologist. A Structured Clinical Interview for DSM (SCID) indicated the panic disorder (DSM-V 300.01). During the interview, the patient emphasized that he became particularly concerned about his and his family’s health since the outbreak of the COVID-19 pandemic. He was also afraid of forced isolation and of employment loss. After the official announcement of the first COVID-19 case in Poland (March 2020), the panic attacks intensified, appearing unexpectedly once a day on average. Despite his deep concerns, the patient had adequate results in reality testing. During the interview, an extended psychological workup was also carried out. The patient’s sensitivity to anxiety was quantified according to the MMPI-2 (Minnesota Multiphasic Personality Inventory). The evaluations of the patient’s anxiety level were performed using the State-Trait anxiety inventory (STAI) developed by Spielberger in the Polish adaptation. Additionally, the X-1 scale was used to register changes in the severity of anxiety. Both the level of state anxiety (current state of anxiety) and trait anxiety measured at the start of treatment were at a high level. The state anxiety (X1) score was 71, and the severity of trait anxiety (X2) score was 67.

Additional analysis of his general and psychological condition included analysis of the exams (EEG record/laboratory blood test) carried out previously, which also did not deliver any hint about any potential physical condition underlying his complains or being the underlying cause for altered state of mind. 

In addition, a miniQEEG exam, targeted on the central strip recordings (C3 and C4 vs Cz), was performed as a baseline for the neurofeedback treatment plan and for the follow-up exams. After completing the diagnostic workup, neurofeedback training was planned and conducted systematically during the two consecutive months. 

During QEEG-neurofeedback sessions, the international 10–20 measurement system was used. Elmiko equipment and DigiTrack software version 12 were used in the study. The amplitude of QEEG rhythms was calculated using Digi Track hardware with the standard producer’s setting. The spectrum of a signal represents the subsets of recordings categorized according to the frequency of the EEG waves. For the mathematical analysis, the algorithm FFT was used, with the results of the function: *f*(z) = A(z) + *j* × F(z). The results of the spectrum analysis in FFT panel in DigiTrack is demonstrated as amplitudes or amplitudes peak-to-peak changes, enabling an easy comparison of the results from any given neurofeedback session with the results from the previous recordings or with the control. In the current analysis, beside the intrinsic comparison (records of study subject, obtained at different time points), an extrinsic healthy control subject was also introduced. Here, a sex-, age- and education-level-matched subject was identified and his records were chosen as a baseline for parallel comparison. Regarding the healthy control, in order to rule out the possible impact of current COVID-19 infection or any other medical condition, an appropriate negative SARS-CoV2 was obtained and previous medical history without any pathological findings was verified.

For the purpose of the current study, miniQEEG and its variability was recorded and documented in concordance with the well-established Sterman’s norms. The initial elimination of artifacts from the EEG recording was performed with the automated software provided by the producer, followed by a manual screening and deletion of remaining artifacts (usually, removal of only several seconds of the whole recording was required).

## 3. Results

### 3.1. Standard Recordings as Reference

According to the standards for QEEG Neurofeedback, a normal QEEG recording is performed on an adult patient at rest, with open eyes, and the following patterns should be observed with the principle: the lower the frequency, the higher the amplitude (Delta less than 20 µV, Theta lower less than 15 µV, Alpha less than 10 µV, sensimotor rhythm (SMR), Beta1 and Beta2 within 5–10 µV). The fast waves (Beta1 and Beta2) are usually attributed to the impact of sympathetic activity on the brain function (stress/excitation), Alpha waves with regular cortical activity/cognitive processes, while Theta activity is assumed to be related to the state of calmness, sleep and activity of the limbic system. The amplitudes of fast waves—Beta1 and Beta2 in the dominant hemisphere—should be higher (up to 50%) compared to those registered in the non-dominant hemisphere [24,25]. Further, in the non-dominant hemisphere, higher amplitudes for slow waves—Delta, Theta, Alpha and SMR—should be present. In the normal situation, in a subject keeping his/her eyes open and remaining at rest, the approximate percentage of waves is as follows: Delta (29%); Theta (22%); Alpha (18%); SMR (13%); Beta1 (9%); Beta2 (9%) (Table 1).

### 3.2. Analysis of the First Mini QEEG Recording (April 2020)

In the case described (right-handed subject), the left hemisphere was assumed to be dominant.

Classic EEG performed and assessed before study inclusion by the clinical neurologist revealed no abnormalities. Notably, the miniQEEG analysis demonstrated an entirely different pattern of electrical brain function, the interpretation of which was made difficult by several irregularities.

First, amplitude distribution over the dominant hemisphere demonstrated some tendency of decrease in the amplitudes associated with the increase in the frequency from Delta to SMR. However, the ratio of Alpha wave amplitudes was more remarkable, particularly when compared to the similar percentage of SMR wave amplitudes. Analysis of the Beta1 amplitudes (C3—5.81 µV and C4—5.09 µV) demonstrated that those amplitudes were almost equal to those of the Alpha waves. This represented a clear abnormality when compared with the standard spectrum: under physiological conditions, Alpha wave amplitudes should be higher than the SMR and Beta1 wave amplitudes.

**Figure 1:**
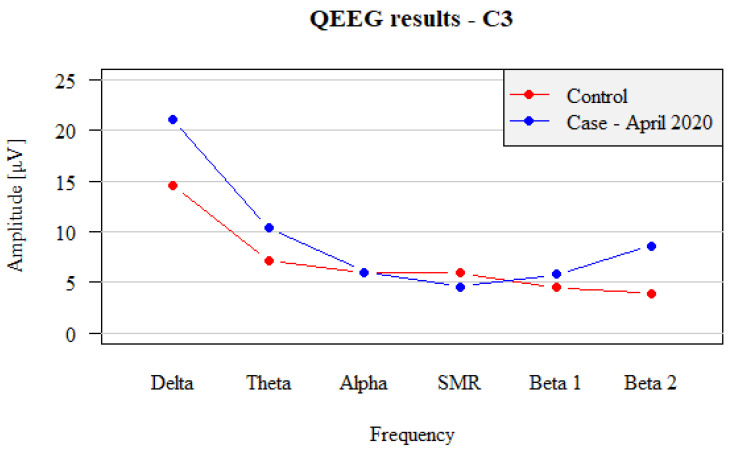
Comparison between left hemispheric (C3) QEEG recordings in test patient with panic attacks (blue dots and lines) before the treatment vs. non-affected subject (control, red dots and lines).

The Beta2 wave amplitude was 8.62 µV (C3) and 8.79 µV (C4), so the Beta2 wave amplitude approached the Theta wave amplitude. By this means, Theta-waves also demonstrated a clear abnormality—according to the standards, the Theta amplitude should be about twice the Beta2, and in the obtained recording, these two groups exhibited almost the same amplitude. As to the SMR pattern: the dominant hemisphere demonstrated the normal decrease in Theta wave amplitudes in relation to Delta and Alpha waves in relation to SMR. SMR wave amplitudes were apparently close to the Alpha wave amplitude. However, instead of equalizing or being lower than the SMR wave amplitudes, Beta1 wave amplitudes visibly increased, achieving the power similar to Alpha wave amplitudes, and Beta2 wave amplitudes increased to the level of Theta wave amplitude (Figure 1).

In the percentage analysis, the difference between Delta, Alpha and Beta2 was most noticeable, similar to results of wave amplitude analysis, presented above.

**Figure 2:**
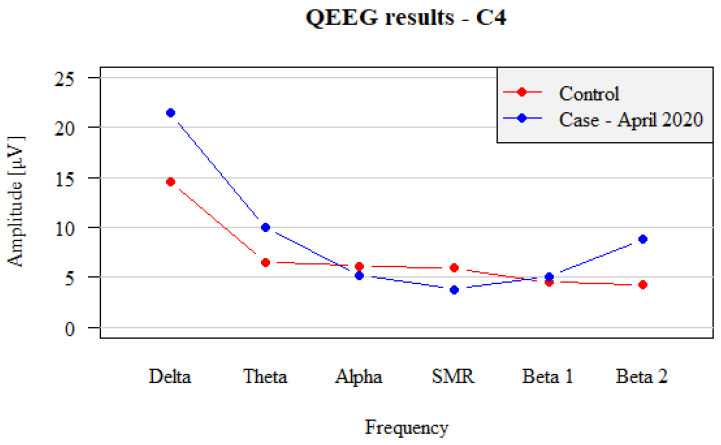
Comparison between right hemispheric (C4) QEEG recordings in test patient with panic attacks before the treatment (blue dots and lines) vs. non-affected subject (control, red dots and lines).

Separate analysis of the QEEG records obtained from the non-dominant hemisphere demonstrated almost identical pattern of changes as in the dominant hemisphere (Figure 2):

In the non-dominant hemisphere, a normal tendency of decreasing amplitudes with increasing frequency from Delta to SMR was observed (Delta 21.44 µV, Theta 10.02 µV, Alpha 5.19 µV and SMR 3.82 µV). In the right-sided recordings, the Alpha wave amplitudes were similar to SMR waves. This trend was the same in the dominant hemisphere. Beta1 amplitudes (5.81 µV) were greater than SMR by about 1.5 µV, and they exceeded the amplitudes of Alpha waves. The Beta2 wave amplitude was 8.62 µV, so the Beta2 wave amplitude was higher than the Alpha wave and close to the Theta wave amplitude. This disturbance was analogous to the abnormality observed for the dominant hemisphere.

In summary, an analysis of the patient’s wave amplitudes in the non-dominant hemisphere demonstrated an expected decrease in Theta wave amplitudes in relation to Delta and Alpha waves in relation to SMR waves, as compared to normal values. SMR wave amplitudes were apparently close to the Alpha wave amplitude. However, Beta1 wave amplitudes increased, reaching the power similar to that of Alpha wave amplitudes (instead of equalizing or being lower than SMR wave amplitudes), and Beta2 wave amplitudes increased to the level of Theta wave amplitudes.

In addition, not only the amplitude pattern, but also the percentage of waves in the recording from the non-dominant hemisphere of the patient, compared to the standard, was characterized by several abnormalities:

Percentages of waves recorded in the patient in C4: Delta 38.3%; Theta 18.5%; Alpha 10.4%, SMR 8.5%; Beta1 10.7%; Beta2 14.7%.

In the percentage analysis, the difference in the Delta, Alpha and Beta2 shares was most prominent, as was the case in the dominant hemisphere. The abnormalities in both hemispheres were analogous.

### 3.3. The Methods and Purpose of QEEG-Based Neurofeedback Training

The patient was subjected to QEEG Biofeedback therapy that was designed based on the results obtained previously during the initial, diagnostic session of miniQEEG. After analyzing the results, the SMR/Theta and Beta2 protocols were initially selected and targeted for neurofeedback training sessions for both hemispheres. The aim of these sessions was both to modulate the QEEG pattern by means of therapeutic action and to document the impact of the treatment on the electrical brain activity over time.

The training sessions were performed on points C3 and C4, for 30 min, with 15 min feedback on each point, until obtaining the first positive results. The treatment units lasted from 1 to 3 min. The aim of the training was to maintain the SMR wave amplitude and to reduce the Beta2 wave amplitude to the value of the SMR wave amplitude. EEG-neurofeedback uses amplification and inhibition of wave amplitudes. In the analyzed case, the waves were inhibited and amplified according to the classic SMR/Theta protocol. The SMR wave was to be amplified. We assumed that the threshold would maintain the initially measured amplitude of about 5 µV. For this reason, we decided that the threshold will not be raised above 4.5 µV. During training, the Theta and Beta2 wave amplitudes were inhibited and the threshold for inhibiting the Theta amplitude was not set lower than 15 µV. In this case, the inhibition of Theta wave amplitudes was aimed at maintaining Theta activity at the same level and monitoring and preventing possible amplification of this wave. The threshold for inhibiting the amplitude of Beta2 waves decreased from a peak of activity—from about 9.5 µV to 6.5 µV. The threshold was decreased proportionally to the decreasing share of this wave. A simple special board platform was chosen to work with the patient. An important issue of training was the patient’s education to carefully follow his own neurophysiological activity—in this case, slow waves (Theta) and fast waves (Beta2). During the first training sessions, each lasting about 30 min, the patient was educated to identify those activities and to attempt to influence their propagation.

### 3.4. Results after Neurofeedback Trainings

**Figure 3:**
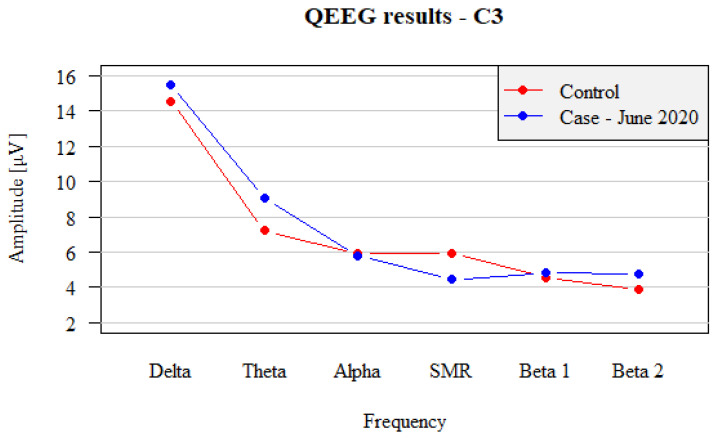
Comparison between left hemispheric (C3) QEEG recordings in test patient with panic attacks after completing the neurofeedback training (blue dots and lines) vs. non-affected subject (control, red dots and lines).

**Figure 4:**
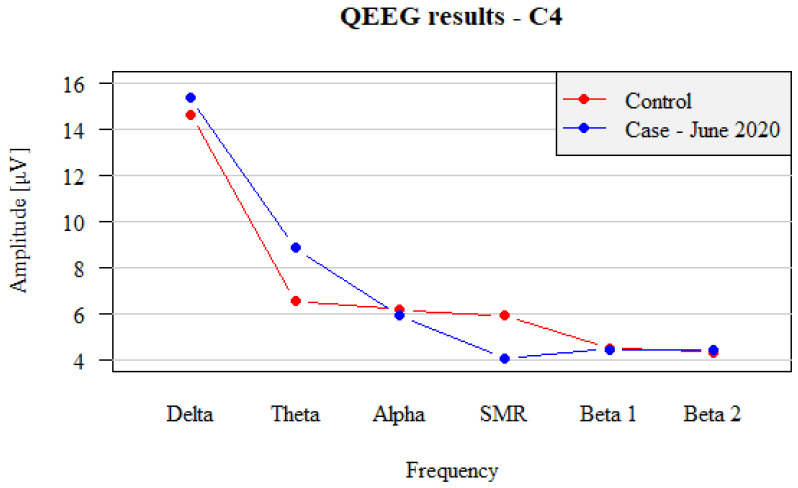
Comparison between right hemispheric (C4) QEEG recordings in test patient with panic attacks after completing the neurofeedback training (blue dots and lines) vs. non-affected subject (control, red dots and lines).

After eight weeks of the management consisting of sixteen neurofeedback training sessions using the SMR/Theta training protocol (twice a week, each lasting 30 min), a favorable shift in the QEEG pattern was observed, with QEEG profile being nearly close to the normal values. The recordings demonstrated some notable changes. The share of Beta 2 waves in both hemispheres decreased from 8.62 µV and 8.79 µV (C3 and C4) to 4.78 µV and 4.41 µV (C3 and C4), respectively. The Alpha wave amplitude also increased, becoming higher than the Beta2 waves. The Theta protocol (inhibition of Theta waves at the level of 15 µV) reduced the fraction of Delta waves from 21 µV to 15.5 µV. A comparison of the records from left hemisphere (C3) after neurofeedback training between the study subject and healthy control is shown in Figure 3, and the right-sided records (C4) in Figure 4.

The results obtained after neurofeedback training suggest that, by means of emotions, the patient has clearly calmed down, was able to sustain the state of calmness and to limit the process of self-excitation. The equalization of SMR, Beta1 and Beta2 wave amplitudes demonstrated the normal process of inhibition of stimuli. The reduced amounts of Delta amplitudes also suggest an increase in cortex activity and normalization of stimulation and rest processes, due to the decrease in the activity of Delta and Beta2 waves. In addition, the Theta activity regained its power. The detailed compilation of the shift in amplitudes of QEEG waves before and after neurofeedback treatment is demonstrated in Table 2. These positive results after two months of intense neurofeedback treatment have proved that the patient understood both the purpose and his task during the training. In addition, the patient himself declared that, at the end of the treatment, he was clearly able to distinguish between neurophysiological status—tension and relaxation—and that he was able to use self-regulation techniques.

### 3.5. Further Results—MiniQEEG Diagnosis and Subjective Effect of Treatment in April 2021 and in April 2022

After 1 year and 2 years, a miniQEEG follow-up examination was performed with the patient. Without any additional training performed in between, the improvement was maintained. The results of the mini QEEG are presented in Table 3. Table 3 contains the compilation of the recorded results of QEEG testing with comparison before initiation of treatment and 2 years after completing the training.

The therapy applied to a patient with panic attacks resulted also in sustained improvement of his cognitive and executive functions. The patient’s anxiety and mood disorders were reduced by strengthening the motivation. This was documented by the query performed according to the STAI questionnaire at all follow-up time points. The results of the questionnaire are presented in Table 4.

## 4. Discussion

In our feasibility study, we were able to subject the patient with COVID-19-related panic disorder to a thorough electrophysiologic workup, using QEEG principles and, based on the initial records—to plan and to perform QEEG-neurofeedback treatment, which provided a long lasting therapeutic effect. This beneficial impact of neurofeedback has been documented not only based on questionnaire-based and subjective self-reporting but also in objective records of follow-up QEEG.

As to the interpretation of the initial QEEG, our findings are not far from the canonical interpretation of wave pattern and are consistent with previous reports on the use of QEEG in diagnosis of anxiety and related disorders [12,18,21,22]. The abundance of Beta1 waves and its relatively high amplitude could be attributed to the state of persistent sympathetic hyperactivity as seen in the condition of the psychical stress. In addition, the equalization of Theta and fast waves (i.e., Beta1 and Beta2) would suggests an extremely strong, abnormal activation of the cerebral cortex, since Theta waves used to be associated with the subcortical limbic system. Thus, the reduced ratio of Theta amplitudes in relation to other waves in our subjects can be interpreted as evidence of the inability of limbic self-regulation in the condition of permanent fear. Such a situation is likely to result in a formation of strong negative emotions (here anxiety) with a total inability to inhibit them by the cerebral cortex. Improvement of the Alpha and Theta wave ratio in relation to the fast waves after therapeutic sessions could be interpreted as regaining the ability of conscious control over the (irrational or exaggerated) fears and concerns.

Previous analyses of QEEG in anxiety patients are consistent with our observations. In patients affected by fear-related conditions including PA, a higher amplitude of low frequency waves (Delta/Theta) accompanied by a relative decrease in the Beta spectrum has been documented [26], clearly differing from the Alpha pattern in healthy subjects [27]. Change in the Alpha spectrum was also the unique attribute of PA-affected QEEG. Here, the fear/anxiety potentially intensifies the non-specific information processing and disturbs the physiological alertness with consecutive Alpha-1 desynchronization [28]. The particularly apparent phenomenon of Alpha suppression by anxiety was documented in the frontal areas [29]. Our study, due to neurofeedback methodology, was limited to recordings from the central strip, distant from the frontal association cortex. However, some similarities with previously reported pattern of anxiety-related QEEG changes may still be described. For example, previously reported hyperexcitability caused by computer simulation of the factor that precipitates fear and reflected by increased absolute Beta power [30], could also be seen in our patient before neurofeedback therapy was initiated. Importantly, our observation of reduced interhemispheric asymmetry as seen in baseline QEEG, was previously documented in a population affected by COVID-19-related anxiety [30,31]. Before the treatment was set, we have also seen higher ratio of slow waves (Delta and Theta) with a significant drop of Alpha power and remarkable increase in Beta2 activity. This observation is not far from the previous reports on anxiety-related QEEG abnormalities. In the study of de Carvalho et al. exposure to the visual simulation, designed to provoke anxiety spells, resulted in the reduction in Alpha wave intensity in the frontal cortex (records from F7, F8, Fp1 and Fp2) in a PD/agoraphobia group. In addition, higher Beta power, related to increased irritability and anticipatory fear, was noted. Similar to our data, reduced absolute Alpha wave power with increased Beta power was recorded during phases of exacerbated fear [30,32]. In addition, Wise et al. documented a similar pattern of QEEG changes in patients burdened by fear (mostly agoraphobia) using 16 channel EEG records. Again, reduced Alpha-1 spectral power, attributed to reduced awareness and to increased processing of non-specific information was recorded. Importantly, these EEG changes were associated with vegetative disturbances, including increased mean heart ratio and impaired skin conductance response [33]. Other results were provided by Hanaok et al. with PD patients, as compared with healthy subjects, who manifested lower coherence values with significant differences in F3–F4, C3–C4, P3–P4, F7–T5 and F8–T6 recordings and the intensity of these changes correlated well with disease duration and the severity of panic attacks [34]. In turn, Knott et. al. reported greater total absolute power in the Delta, Theta, and Alpha bands, and lower relative power in the Beta band in PD subjects, as compared with healthy controls. Interestingly, the absolute power of Delta and Theta correlated well with the rating of anxiety attributed by independent observers, while relative Beta power was associated with the self-esteem of anxiety [35]. Certainly, the behavioral and emotional changes, including panic attacks, may result from an organic disease of the central nervous system and the question arises, as to whether the infection with SARS-CoV2, as a neurotropic virus, may result in such disturbances and if QEEG may be helpful in a differential diagnostic workup. An interesting analysis was provided by Dantendorfer et al. Here, the structural abnormalities in the brains of PD patients were documented or excluded by magnetic resonance imaging and the possibility of their detection with the use of EEG was assessed. Indeed, QEEG was successful in identifying the PD subjects, carrying the morphological abnormalities [36].

As to the potential causative role of SARS-CoV2 infection for the anxiety-related disorders, we believe, that in the majority of cases, COVID-19 causes panic attacks not by infection itself but via the constant exposure to negative information. In this matter we share the opinion of Fiorillo et al., who demonstrated an exponentially increased number of individuals affected by psychopathological changes, not necessarily due to direct contact with the virus [37]. On the other hand, patients affected by anxiety or prone to this disturbance are particularly sensitive to external stimuli, including physical and respiratory ones. Thus, pandemic safety measures, including the mandatory use of face masks, could further exacerbate signs related with airway resistance (dyspnea) [38,39]. Of note, the occurrence of PD cases similar to above cited reports as well as to our case description seemed to snowball. The current socioeconomic changes, including the COVID-19 pandemic, still pose a threat for our society, with sustained feelings of fear/anxiety.

In our case, implementation of neurofeedback treatment was highly and durably efficient in improving both the subjective burden of panic attacks as well as abnormalities seen in initial QEEG. After a series of QEEG biofeedback sessions, increase in Alpha activity with reduction in both Delta/Theta spectrum was recorded in the central strip area. Of note, amelioration of electrical activity was not limited to the time period directly after neurofeedback treatment but was sustained even 2 years after completing the training. As to the subjective assessment, during the 1-year pandemic period, the patient self-reported an improved mood and behavior as well as calmness, patience and lack of negative thoughts. Due to neurofeedback sessions, his panic attacks actually dissolved, so that he was able to enjoy daily life, achieving and keeping a high level of psychological relief. According to his personal evaluation, neurofeedback sessions could be described as pleasant and easy to manage for virtually everyone. Facing the continuous and long-lasting improvement, both in STAI questionnaire as well as in QEEG records up to 2 years after completing the treatment, we share his point of view; in our opinion, QEEG Neurofeedback is a useful therapeutic tool, ready to be implemented in the vast population of COVID-19-related anxiety patients.

## 5. Limitations

Certainly, our study is not free from limitations. The paper describes a single case observation and a single neurofeedback protocol. Thus, further analyses using different neurofeedback programs and hardware would be necessary to ascertain the real therapeutic potential. However, our analysis was intended as a feasibility test, documenting the possibility of implementing a simple and cost-effective miniQEEG analysis on patients with COVID-19-related panic attacks and to document the general usefulness of this method, both for diagnostic and therapeutic purpose. Our report demonstrates the effectiveness of neurofeedback use only in line C3-Cz-C4 with the choice of SMR/Theta protocol. Potentially, various patterns of QEEG application may be required for different patients, depending on disease form, severity, duration and—most importantly—on the individual pattern of QEEG changes seen in initial recordings before the treatment is set.

## 6. Conclusions

Neurofeedback therapy based on QEEG recording is feasible and easy to implement in subjects also suffering from anxiety related to the COVID-19 pandemic and other global threats. The positive effects of treatment may be significant and remarkably persisting. The growing number of anxiety-affected individuals create the vast new area of application for QEEG biofeedback.

## Figures and Tables

**Table 1:** Example of normal QEEG record with amplitude standards in the central strip points (C3 and C4 vs. Cz).

Channel		Delta 0.5–3.0 Hz	Theta 4.0–8.0 Hz	Alpha 8.0–12.0 Hz	SMR 12.0–15.0 Hz	Beta 1 15.0–20.0 Hz	Beta 2 20.0–34.0 Hz
C3	Amplitude (µV)	±20 (~20% < C4)	±15 (~20% < C4)	±10 (~20% < C4)	4–10 (~20% < C4)	4–10 (~20% > C4)	4–10 (~20% > C4)
C4	Amplitude (µV)	±20 (~20% > C3)	±20 (~20% > C3)	±20 (~20% > C3)	4–10 (~20% > C3)	4–10 (~20% < C3)	4–10 (~20% < C3)

**Table 2:** Comparison of QEEG results from April 2020 (before neurofeedback treatment) and from July 2020 (directly after completing the neurofeedback treatment program).

Channel		Delta 0.5–3.0 Hz		Theta 4.0–8.0 Hz		Alpha 8.0–12.0 Hz		SMR 12.0–15.0 Hz		Beta 1 15.0–20.0 Hz		Beta 2 20.0–34.0 Hz	
	*Year*	*2020*	*2022*	*2020*	*2022*	*2020*	*2022*	*2020*	*2022*	*2020*	*2022*	*2020*	*2022*
C3	Amplitude (µV)	21.06	14.46	10.44	7.29	6.04	5.26	4.59	4.16	5.81	4.68	8.62	4.34
C4	Amplitude (µV)	21.44	14.53	10.02	7.98	5.19	6.34	3.82	4.25	5.09	4.17	8.79	4.83

**Table 3:** Comparison of QEEG results from April 2020 (before neurofeedback treatment) and from April 2022 (2 years after completing the neurofeedback treatment).

Channel		Delta 0.5–3.0 Hz		Theta 4.0–8.0 Hz		Alpha 8.0–12.0 Hz		SMR 12.0–15.0 Hz		Beta 1 15.0–20.0 Hz		Beta 2 20.0–34.0 Hz	
	*Year*	*2020*	*2022*	*2020*	*2022*	*2020*	*2022*	*2020*	*2022*	*2020*	*2022*	*2020*	*2022*
C3	Amplitude (µV)	21.06	15.5	10.44	9.05	6.04	5.8	4.59	4.45	5.81	4.86	8.62	4.78
C4	Amplitude (µV)	21.44	15.31	10.02	8.85	5.19	5.9	3.82	4.05	5.09	4.46	8.79	4.41

**Table 4:** Baseline and follow-up scoring of anxiety in study subject during the prolonged observation of the treatment results.

State-Trait Anxiety Inventory (STAI) the X-1 Scale	
First interview	71 scores/10 sten
After 8 weeks/16 EEG Biofeedback trainings	44 scores/6 sten
After 1 year (2021)	41scores/6 sten
After 2 years (2022)	42 scores/6 sten

## Data Availability

The datasets generated during and/or analyzed during the current study are available from the corresponding author on reasonable request. Compliance with Ethical Standards Conflict of Interest On behalf of all authors.
